# Comparative treatment costs of risk‐stratified therapy for childhood acute lymphoblastic leukemia in India

**DOI:** 10.1002/cam4.5140

**Published:** 2022-08-15

**Authors:** Tushar Mungle, Nandana Das, Saikat Pal, Manash Pratim Gogoi, Parag Das, Niharendu Ghara, Debjani Ghosh, Ramandeep Singh Arora, Nickhill Bhakta, Vaskar Saha, Shekhar Krishnan

**Affiliations:** ^1^ Clinical Research Unit Tata Translational Cancer Research Centre, Tata Medical Center Kolkata India; ^2^ Tata Consultancy Services Tata Translational Cancer Research Centre, Tata Medical Center Kolkata India; ^3^ Department of Paediatric Haematology and Oncology Tata Medical Center Kolkata India; ^4^ Department of Medical Oncology Max Super Speciality Hospital New Delhi India; ^5^ Department of Global Pediatric Medicine St Jude Children's Research Hospital Memphis Tennessee USA; ^6^ Division of Cancer Sciences, Faculty of Biology, Medicine and Health School of Medical Sciences, University of Manchester Manchester UK

**Keywords:** cost‐effectiveness, low‐middle income countries, pediatric acute lymphoblastic leukemia, risk‐stratified therapy, treatment cost

## Abstract

**Background:**

To evaluate the treatment cost and cost effectiveness of a risk‐stratified therapy to treat pediatric acute lymphoblastic leukemia (ALL) in India.

**Methods:**

The cost of total treatment duration was calculated for a retrospective cohort of ALL children treated at a tertiary care facility. Children were risk stratified into standard (SR), intermediate (IR) and high (HR) for B‐cell precursor ALL, and T‐ALL. Cost of therapy was obtained from the hospital electronic billing systems and details of outpatient (OP) and inpatient (IP) from electronic medical records. Cost effectiveness was calculated in disability‐adjusted life years.

**Results:**

One hundred and forty five patients, SR (50), IR (36), HR (39), and T‐ALL (20) were analyzed. Median cost of the entire treatment for SR, IR, HR, and T‐ALL was found to be $3900, $5500, $7400, and $8700, respectively, with chemotherapy contributing to 25%–35% of total cost. Out‐patient costs were significantly lower for SR (*p* < 0.0001). OP costs were higher than in‐patient costs for SR and IR, while in‐patient costs were higher in T‐ALL. Costs for non‐therapy admissions were significantly higher in HR and T‐ALL (*p* < 0.0001), representing over 50% of costs of in‐patient therapy. HR and T‐ALL also had longer durations of non‐therapy admissions. Based on WHO‐CHOICE guidelines, the risk‐stratified approach was very cost effective for all categories of patients.

**Conclusions:**

Risk‐stratified approach to treat childhood ALL is very cost‐effective for all categories in our setting. The cost for SR and IR patients is significantly reduced through decreased IP admissions for both, chemotherapy and non‐chemotherapy reasons.

## Graphical abstract

With the ICiCLe‐ALL‐14 risk stratified approach, ALL treatment cost for low‐risk patients is significantly lower as compared to intermediate and high‐risk groups. Additionally, it is a cost‐effective intervention in a low‐middle income country.

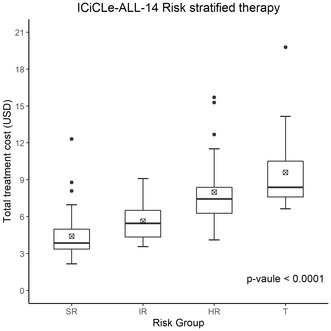

## 1 INTRODUCTION

The WHO Global Initiative on Childhood Cancer aims to reach a 60% survival rate for children with cancer by 2030 by doubling the global cure rate of the six most common cancers with high cure rates.^1^ Childhood cancer is not on the priority list of the Indian National Programme for Prevention and Control of Cancers, Diabetes, Cardiovascular Diseases, and Stroke, or the National Cancer Control Programme. A commonly reported rationale is the high burden of communicable diseases and costs of treating children with cancer beyond available resources.^2^, ^3^ On the contrary, as the burden of communicable diseases decreases, deaths due to cancer contribute significantly to childhood mortality in low‐middle income countries (LMIC). Particularly, in India, there has been a rapid decline in childhood deaths due to communicable diseases.^4^ Annually, an estimated 76,805 new cases of cancer occur in the under 19‐year‐olds in India. Of these, 20,716 have leukemia of which around 15,000 are acute lymphoblastic leukemia (ALL).^5^ In high‐income countries, outcomes for childhood ALL now approaches 90%.^6^ Contrastingly, in India, the outcomes at specialized centers have remained at around 65%–70% for the last three decades. A variety of protocols without risk stratification have been used, with all children receiving full intensity therapy with treatment‐related deaths of 11%–26% and 15%–41% relapse rates.^7^, ^8^, ^9^

In order to reduce deaths and relapse rates, in 2013, the Indian Childhood Collaborative Leukemia (ICiCLe) Group launched a multicenter open label risk‐stratified randomized clinical trial in childhood ALL. The aims of the research study were to (i) standardize therapy for ALL across centers so that patients could be reassured that they need not travel to other centers (ii) decrease intensity of therapy to reduce cost and toxicity. The trial is ongoing and the impact on toxicities and outcomes are awaited. As an implementation outcome associated with the clinical trial, we report the cost and cost effectiveness of the risk‐stratified treatment strategy at a single center—Tata Medical Center (TMC), Kolkata, India.

## 2 PATIENTS AND METHODS

### 2.1 Patients and hospital

TMC is a charitable not‐for‐profit tertiary care cancer center with a dedicated pediatric cancer unit, inclusive of inpatient (IP), outpatient (OP), day‐care and a free residential stay facility for poor families. Children (1–18 years), who began and completed treatment for ALL at TMC, between August 2013 and March 2019 were included in this study. Those who had an event, defined as death, relapse or treatment abandonment prior to completion of therapy, were excluded from the study.

### 2.2 Treatment

All children were recruited and treated on the ICiCLe‐ALL‐14 clinical trial (CTRI/2015/12/006434) after consent.^10^ Participating centers in this trial are the Tata Memorial Hospital, Mumbai, Postgraduate Institute of Medical Education and Research, Chandigarh, All India Institute of Medical Sciences, New Delhi, Cancer Institute, Chennai and TMC. Treatment is split into five blocks—induction, consolidation, interim maintenance, delayed intensification and maintenance. Children were risk‐stratified on day 8. B‐cell precursor ALL (BCP‐ALL) with a good prednisone response (<1000 peripheral blasts/μl at day 8), non high‐risk cytogenetics, and CNS 1–2 were treated as standard risk (SR) if age was 1 to <10 years and presenting white cell count <50 × 10^9^/L in the absence of organomegaly; and as intermediate risk (IR) if age ≥10 to <19 years or presenting white cell count ≥50 × 10^9^/L as well as SR patients with organomegaly. BCP‐ALL with high‐risk cytogenetics or CNS3 or prednisolone poor response (≥1000 peripheral blasts/μl at day 8) were treated as high risk (HR). T‐ALL was treated separately, but similar to HR. BCP‐ALL SR and IR with minimal residual disease (MRD) of ≥10^−4^ at the end of induction were subsequently treated as HR. Through induction, consolidation and interim maintenance, SR patients received reduced intensity therapy aimed at decreasing toxicity; moderately increased for IR with full intensity therapy for HR and T‐ALL. All patients received the same delayed intensification and maintenance therapy (Table S1).^10^

All patients were admitted when first diagnosed and discharged as soon as clinically well. Further therapy for SR and IR patients was day‐care/OP‐based while HR and T‐ALL patients were admitted for high dose methotrexate infusions. Patients were admitted for complications of therapy, while blood and blood products were administered in day‐care.

### 2.3 Data source

The hospital has an electronic medical record (EMR) and billing system. Patients are categorized as general or private and only those in the general category (90% of patients) were included in this analysis. Billing data are available in separate sections for OP, day‐care and IP stays, as well as costs of hospitalization, investigations, drugs, fluids, and consumables. The billing data contain all expenditures incurred by the patient and include charges for interventions and OP pharmacy.

### 2.4 Assessment of treatment costs

The predicted cost of chemotherapy alone for each risk category, based on a reference surface area of 1 m^2^, was obtained from the hospital pharmacy. The actual treatment and procedures' cost for each individual was obtained from the itemized billing data and separated into costs for IP and OP/day‐care episodes. Patients' electronic hospital records were analyzed to obtain the duration of IP admissions and to distinguish IP admissions for chemotherapy from those related to management of complications. For non‐chemotherapy‐related IP admissions, IP costs for first admission (induction) and high dose methotrexate infusions were excluded. All other phases of therapy were managed through OP/day‐care. Demographic data and distance traveled to the hospital from their postal address were extracted from the EMR.

### 2.5 Disability‐adjusted life years

Disability‐adjusted life years (DALYs) were calculated using previously published methods.^11^, ^12^ The model assumptions and values relevant to this study are described in Table S2. Cost effectiveness was defined using the WHO‐CHOICE criteria,^13^ with treatment considered to be very cost effective if the cost per DALY averted was less than 1 GDP, and cost effective if the intervention is between 1 and 3 GDP. As the analyses included only patients who had completed therapy, lost‐to‐follow up was not included in the analysis. Late effects, rare in childhood ALL, have not been included in the model.

### 2.6 Statistical analysis

Data were checked for normality visually with Q–Q/density plot and statistically with Shapiro–Wilk test. Kruskal–Wallis and/or Mann–Whitney test were used to test for differences in overall, IP and OP/day‐care costs. Paired risk group analyses were performed using pairwise Wilcoxon test. All statistical analyses were performed using R (version 3.6.1). Costs are represented in United States Dollars ($) with 1$ = ₹65.27. This is the average of the exchange rates from the date of first patient enrolled to the treatment completion date of the last enrolled patient.

## 3 RESULTS

Treatment costs for the entire duration of treatment were analyzed in 145 patients—50 SR, 36 IR, 39 HR, and 20 T‐ALL. Patient characteristics are described in Table 1.

**TABLE 1:** Demographic data of patients included in the study

	Total	SR	IR	HR	T	*p*‐value
*N* (%)	145	50 (35)	36 (25)	39 (27)	20 (14)	
Age (years)						0.0071
Median	5	4.25	5.28	5.37	8.66	
IQR	3.30–7.74	3.1–6.1	3.4–10.8	3.2–7.2	4.6–12.3	
Mean (*SD*)	6.1 (3.7)	4.7 (2.0)	6.7 (4)	6 (3.7)	8.8 (4.8)	
Sex (%)						
Male	88 (61)	31 (62)	20 (56)	22 (56)	15 (75)	
Female	57 (39)	19 (38)	16 (44)	17 (44)	5 (25)	
Lineage (%)						
BCP‐ALL	125 (86)	50	36	39	–	
T‐ALL	20 (14)	–	–	–	20	
Distance traveled (km)						0.1278
Median	118	104^a^	172	124	38	
IQR	29–295	25–185	47–433	36–298	21–395	
Mean (*SD*)	167 (218)	156 (185)	243 (218)	221 (235)	200 (252)	

Abbreviations: ALL, acute lymphoblastic leukemia; HR, high risk; IQR, interquartile range; IR, intermediate risk; SD, standard deviation; SR, standard risk.

^a^ For one SR patient distance could not be computed.

Chemotherapy costs for a patient with body surface area of 1 m^2^ is estimated to be $1420, $1690, $1985, and $2117 for SR, IR, HR, and T‐ALL, comparable to that previously reported in Delhi.^14^ It represents 120%, 140%, and 150% increase in costs over SR, respectively (Table S3). For SR, IR, and HR, the most expensive phase of treatment is induction, while for T‐ALL it is interim maintenance. The median cost of treatment was $3900, $5500, $7400, and $8700, for SR, IR, HR, and T‐ALL, representing 141%, 190%, 223% excess costs over SR, respectively (Table 2). Thus, while the treatment cost differs significantly between each risk group, the chemotherapy cost contributes to only 25%–35% of total costs. To investigate this further, we looked separately at costs incurred for OP and IP therapy.

**TABLE 2:** Cost of treatment in the different risk categories

	SR	IR	HR	T
Overall costs				
Median (IQR)	3.9 (3.4–5.0)	5.5 (4.2–6.7)	7.4 (6.2–9.1)	8.7 (7.6–10.8)
Mean (*SD*)	4.4 (1.8)	5.6 (1.5)	8.5 (3.9)	10.2 (4.2)
*p*‐value				
SR	–	<0.0001	<0.0001	<0.0001
IR	–	–	<0.0001	<0.0001
HR	–	–	–	0.0165
OP costs				
Median (IQR)	2.4 (2.1–2.7)	3.4 (2.9–3.8)	3.5 (3.1–4.1)	3.8 (3.1–4.5)
Mean (*SD*)	2.5 (0.6)	3.4 (0.6)	3.8 (1.4)	3.8 (0.9)
*p*‐value				
SR	–	<0.0001	<0.0001	<0.0001
IR	–	–	0.1706	0.042
HR	–	–	–	0.4794
IP costs				
Median (IQR)	1.6 (0.9–2.4)	2.1 (1.5–3.1)	3.7 (3.0–4.9)	5.4 (3.9–6.9)
Mean (*SD*)	2.0 (1.5)	2.3 (1.3)	4.6 (3.2)	6.4 (3.9)
*p*‐value				
SR	–	0.1206	<0.0001	<0.0001
IR	–	–	<0.0001	<0.0001
HR	–	–	–	0.0059

*Note*: Costs are given in USD ($)/1000.

Abbreviations: HR, high risk; IP, inpatients; IQR, interquartile range; IR, intermediate risk; OP, outpatients; SD, standard deviation; SR, standard risk.

Outpatient costs were significantly lower for SR compared to all other groups (*p* < 0.0001). IR, HR and T‐ALL patients receive infusions of daunorubicin and cyclophosphamide in OP/day care, and OP/day care costs were comparable in all these three categories. OP costs were higher than IP costs for both SR and IR. In HR, IP and OP costs were comparable but IP costs were considerably higher in T‐ALL (Table 2). Non‐SR patients may have been more frequently transfused with blood products adding to day care costs, but this was not investigated. HR and T‐ALL patients also receive anti‐fungal prophylaxis with intravenous infusions of liposomal amphotericin in induction and consolidation adding to the increase in OP/day care costs.

Median IP costs were comparable between SR ($1600) and IR ($2100) but significantly higher for HR ($3700) and T ($5400) (*p* < 0.0001), with mean costs of HR and T‐ALL almost double and triple of that for SR (Table 2). Both HR and T‐ALL are admitted for four fortnightly high dose methotrexate infusions in interim maintenance, each lasting an average of 3‐days. The dose of methotrexate is 3 and 5 g/m^2^ for HR and T‐ALL, respectively, so the drug costs are 1.5 times higher for T‐ALL (Table S3). Nevertheless, the admissions' cost and high dose methotrexate alone does not account for the observed increase in IP costs for HR and T‐ALL.

We excluded IP costs for the induction and high dose methotrexate treatment phases to obtain IP costs that only reflect admissions for complications of therapy, primarily for the management of febrile neutropenia. The cost of non‐therapy admissions were comparable in SR and IR, but significantly lower (*p* < 0.0001) than that associated with HR and T‐ALL (Table 3). Median cost of IP admissions for complications of therapy was $700, $1100, $2100, and $2700, or 44%, 52%, 57%, and 50% of IP costs in SR, IR, HR, and T‐ALL, respectively (Table 3). Increasing intensity of therapy in induction and consolidation is associated with a higher incidence of complications and deaths.^15^ In induction, SR patients do not receive any anthracycline. IR patients receive two doses and HR and T‐ALL 4‐doses of daunorubicin (25 mg/m^2^ per dose). In consolidation, IR patients receive BFM Ib^16^ while HR and T‐ALL receive augmented Ib.^17^ The higher intensity of therapy in HR and T‐ALL is associated with a significantly higher number of admissions (*p* = 0.0115), days of hospitalization (*p* = 0.0008) as well as an increase in the average number of days admitted per admission (*p* = 0.0308) (Table 4). Our standard practice for febrile neutropenia is to discharge clinically stable children with sterile blood cultures if afebrile after 48 h of intravenous antibiotics. If still febrile after 48–72 h, antibiotics are changed to cover multidrug resistant bacteria which increases the cost. The longer duration of IP stay in HR and T‐ALL potentially reflects either positive blood cultures or persistent fever.

**TABLE 3:** Cost of non‐chemotherapy‐related inpatient admissions in the different risk categories

	SR	IR	HR	T
Cost				
Median (IQR)	0.7 (0.2–1.5)	1.1 (0.4–1.6)	2.1 (1.5–3.2)	2.7 (1.6–5.0)
Mean (*SD*)	1.1 (1.3)	1.3 (1.1)	2.9 (3.0)	4.0 (4.1)
*p*‐value				
SR	–	0.3916	<0.0001	<0.0001
IR	–	–	<0.0001	<0.0001
HR	–	–	–	0.3356

*Note*: Costs are given in USD ($)/1000.

Abbreviations: HR, high risk; IQR, interquartile range; IR, intermediate risk; SD, standard deviation; SR, standard risk.

**TABLE 4:** Details for number of hospital admissions

	SR	IR	HR	T	*p*‐value
Number of admissions					0.0115
Median (IQR)	3.0 (1–5)	3.0 (2–5)	4.0 (3–6)	5.0 (2.3–7)	
Mean (*SD*)	3.3 (2.3)	3.6 (2.4)	5.0 (2.8)	5.1 (2.8)	
In‐patient days					0.0008
Median (IQR)	9.0 (4–20)	9.5 (4–18)	16.0 (9–24)	22.0 (10–33)	
Mean (*SD*)	12.0 (11)	12 (9)	18.8 (15)	23.7 (17)	
Average days per admission					0.0308
Median (IQR)	3.1 (2–4)	2.7 (2–4)	3.0 (3–5)	3.9 (3–6)	
Mean (*SD*)	3.0 (1.5)	2.9 (1.7)	3.9 (2.2)	5.1 (4.0)	

Abbreviations: HR, high risk; IQR, interquartile range; IR, intermediate risk; SD, standard deviation; SR, standard risk.

The estimated annual burden of childhood ALL in India is 388,602 DALYs. As shown in Table 5 (for calculations please refer to Supplement 2), at a discounting rate of 3%, an estimated 248,706 DALYs representing 64% of the estimated annual disease burden, are averted with contemporary risk‐stratified treatment. In contrast, the estimated impact on disease burden with all‐purpose intensive therapy is 1.5 times lower (148,176 DALYs averted), representing 38% of the estimated annual disease burden. When compared to offering no treatment, the estimated incremental cost‐effectiveness ratio with risk‐stratified treatment (USD 354 per DALY averted) is less than half that with all‐purpose intensive therapy (USD 749 per DALY averted) (Table S4). As described in Table 6, when evaluated against the estimated national threshold for very cost‐effective interventions (USD 20,752), the cost of risk‐stratified treatment (USD 5880 per patient; 17% of cost threshold) is half of that with all‐purpose intensive therapy (USD 7400 per patient: 36% of cost threshold). When analyzed by risk groups, risk‐stratified treatment cost as a proportion of the very cost‐effective intervention threshold is nearly three times lower for SR (8%) and nearly 1.5 times lower for IR (13%) compared to HR and T‐ALL treatment (22% and 27%, respectively).

**TABLE 5:** DALY's and cost‐effectiveness of all‐purpose therapy and risk‐stratified therapy

	All‐purpose therapy			Risk‐stratified therapy		
	3%	6%	0%	3%	6%	0%
DALYs lost per untreated case (years)	25.9	15.82	49.8	25.9	15.7	47.55
National DALYs with no treatment (years)	388.60	237.59	750	388.60	237.59	740
National DALYs averted with treatment (years)	148.18	146.71	463	248.71	190.07	592
Upper limit of cost effective, USD/case	62.26	61.64	64.83	104.49	79.86	248.71
Upper limit of very cost effective, USD/case	20.75	20.55	194.50	34.83	26.62	82.90

*Note*: Costs are given in USD ($)/1000.

Abbreviation: DALY, disability‐adjusted life years.

**TABLE 6:** DALY's and cost‐effectiveness of therapy in the different risk groups

	SR			IR			HR			T		
	3%	6%	0%	3%	6%	0%	3%	6%	0%	3%	6%	0%
DALYs lost per untreated case (years)	26.05	15.86	50.66	25.84	15.81	49.69	25.82	15.81	49.6	25.06	15.63	46.41
National DALYs with no treatment (years)	390.88	238.09	760	387.66	237.38	745	387.36	237.31	744	376.05	234.64	696
National DALYs averted with treatment (years)	338.08	221.43	698	300.21	208.89	647	235.67	185.10	573	228.79	183.02	536
Upper limit of cost effective, USD/case	142.04	93.03	293.17	26.13	87.77	272.03	99.02	77.77	240.68	96.12	76.89	224.99
Upper limit of very cost effective, USD/case	47.35	31.01	97.72	42.04	29.26	90.68	33.01	25.92	80.23	32.04	25.63	75.00

*Note*: Costs are given in USD ($)/1000.

Abbreviations: DALY, disability‐adjusted life years; HR, high risk; IR, intermediate risk; SR, standard risk.

## 4 DISCUSSION

To our knowledge, the study provides the first combined cost and cost‐effectiveness data for children with ALL diagnosed and treated on a prospective clinical trial in an LMIC. Additionally, we sub‐stratify our results to understand how risk‐stratification modulates costs and impacts on cost‐effectiveness ratios. For health policy makers and patient advocates, these data are critical for prioritization discussions and to ensure budgets earmarked for children with ALL are adequately financed.

The median cost of treating a child with ALL at our hospital ranged from $3900 to $8700. This is comparable to costs of $3234–$30,000, using a mixture of western and indigenized protocols, reported by other LMIC's. These protocols have mostly been non‐risk stratified^18^, ^19^, ^20^ or risk‐stratified using clinical and laboratory parameters^21^, ^22^ with one protocol using MRD risk stratification^23^ though the latter omitted HR patients. The combined reported experience suggests that increased intensity of therapy is associated with a higher incidence of morbidity and treatment deaths. Additionally, risk stratification plays an important role in costs. By transparently providing the costs associated with a standardized protocol, clinical teams can begin to understand trade‐offs in toxicity, potential for survival and cost as variables when developing locally relevant clinical practice guidelines. Without risk stratification, all BCP‐ALL patients would need to be treated uniformly which, in our case, would mean decreasing intensity of treatment for HR, potentially increasing relapse rates. At our center, the additional cost of risk stratification in the ICiCle protocol is for fluorescent in situ hybridization screening for genetic risk stratification^24^ and flow cytometry for ploidy and MRD.^25^, ^26^ This costs under $300 per patient. Our results show that the risk stratification used in the ICiCLe protocol, significantly decreases the treatment cost for SR and IR patients. The report on the toxicities in the different risk groups is awaited from the currently open clinical trial^10^ and beyond the scope of this study. Early reported trial‐related mortality has not flagged concerns. The data analyzed here suggests that SR and IR patients were less frequently admitted for treatment related complications and had shorter durations of hospitalization.

Although the results of our study of treating pediatric ALL in the Indian context is cost‐effective, cost‐effectiveness does not equate to affordability.^27^ The per capita income of families in India is around $1900 per annum (for 2020).^28^ Thus, even the cost of SR treatment will be a struggle for many families. Though India's per capita expenditure on health care is around $23 per annum, there are now both national (e.g. Ayushman Bharat Yojna) and a number of state schemes^29^ offering up to $7000 per family per year, moving India toward universal healthcare.^30^ Support for IP treatment for children with cancer comes from government, non‐government (NGO) agencies as well as private donors who pay the hospitals directly. The amounts available are usually adequate to cover IP costs (Table 2) unless there are major complications, though patients may need to initially finance treatment until aid packages are granted. OP costs are not usually covered. However, in SR and IR patients, OP costs are higher than IP costs. Traveling to hospital, overnight stays and often costs of drugs are borne by the families. Out‐of‐pocket expenses (OOPE) have not been calculated in this study. The average distance traveled to our hospital by families is 167 kms. The longer distances traveled increases the OOPE^31^ as the cost of travel and food is compounded by potential loss of income^32^ and the necessity of finding a place to stay.^33^ It is our experience, irrespective of their economic status that families want the best treatment for their children. They travel across the country to the center they perceive provides the best care for their child. OOPE are more severe for rural and low‐income populations^34^ and both, distances traveled to hospital and OOPE costs are implicated in treatment abandonment.

Due to the lack of public financing for childhood cancer services, a number of NGOs have stepped in to bridge this gap. Examples include St Jude India Child Care Centers which provide free of charge care to families of children with cancer throughout India. CanKids also manages “home away from home” facilities and supports poorer families financially as well as providing free medicines. There are other local charities as well as donor programs from pharmaceutical companies. While the results of this study can help advocacy programs align their fundraising goals with existing patient burden projections, these data are also helpful as a mechanism to begin formulating schemes for government budgetary support. As central or state governments demonstrate an interest in supporting childhood cancer through universal health care packages, following the World Health Organization Cure*All* framework recommendations,^35^ then cost and cost‐effectiveness data will be required for implementation.

There are a number of caveats to our interpretations. When calculating costs of IP admissions related to complications of therapy, we excluded the initial admissions for induction for all patients and for high dose methotrexate infusions in HR and T‐ALL. For a few patients, complications occur whilst on therapy and our analyses may underestimate the actual non‐therapy costs incurred. The study focused on the cost of the entire treatment excluding those who did not complete therapy due to death, relapse or abandonment while on treatment. These comprise <10% of patients and we feel are unlikely to significantly influence the analyses. In childhood ALL, around 5% of patients are eligible for allogeneic stem cell transplantation in first remission (CR1). As reported by us previously,^36^ the majority of our patients cannot afford this process and no patient was transplanted in CR1. While risk stratification increased DALY's, we await to see the survival outcomes of the SR/IR group as any increase in relapse rates may overestimate cost‐effectiveness. Our hospital is a private not‐for‐profit center. Costs may differ at other centers where funding is from government, semi‐government or private sources. At times, donors and pharma have provided free drugs for use by poor patients, and patients have pooled resources to buy the more expensive drugs (e.g. polyethylene conjugated l‐Asparaginase). On occasion patients have acquired drugs from outside the hospital. These and OOPE have not been analyzed, making the interventions appear more favorable compared to reality. Furthermore, we have only taken the final post induction risk stratification into account. Costs have not been separately calculated for patients who were initially SR/IR in induction and then treated as HR based on MRD ≥10^−4^ at the end of induction.

The Lancet Oncology commission on sustainable care for children with cancer recommended the incorporation of childhood cancers into essential benefits packages when expanding universal health coverage.^2^ However, as the Seguro Popular program in Mexico demonstrated, providing universal coverage for all children with cancer does not necessarily improve outcomes.^37^ The process should also incorporate evidence‐based practice, ideally through clinical trials or locally adapted clinical practice guidelines. By transparently providing the costs associated with a discernable protocol, clinical teams can begin to understand trade‐offs in toxicity, potential for survival, and cost as variables when developing locally relevant clinical practice guidelines. The National Cancer Grid^38^ is in the process of developing standard guidelines for cancer treatment throughout India and is a funder of the multicenter ICiCLe‐2014 clinical trial. Unlike with the more common communicable diseases of childhood, children with cancer require prolonged financial support without which many families will be driven to long term debt and poverty. Many childhood cancers are curable at costs within the reach of the different healthcare packages that now exist in India. Healthcare providers and funding agencies need to work together to provide a comprehensive financial package covering hospital and OOPE so that all children with cancer in India receive curative therapy.

## AUTHOR CONTRIBUTIONS

**Tushar Mungle:** Data curation, formal analysis, methodology, writing‐original draft and writing‐review and editing. **Nandana Das:** Data acquisition and data curating, formal analysis, writing – original draft and writing‐review and editing. **Saikat Pal:** Data acquisition and data curating, formal analysis. **Manash Pratim Gogoi:** Data curating, writing – review and editing. **Parag Das:** Data curating, writing – review and editing; **Niharendu Ghara:** Data curating, writing – review and editing; **Debjani Ghosh:** Data curating, writing – review and editing; **Ramandeep Singh Arora:** Formal analysis, methodology, writing – review and editing; **Nickhill Bhakta:** Formal analysis, methodology, writing – original draft and writing – review and editing; **Vaskar Saha:** Conceptualization, funding acquisition, formal analysis, methodology, writing – original draft and writing – review and editing. **Shekhar Krishnan:** Conceptualization, funding acquisition, formal analysis, methodology, writing – original draft and writing – review and editing.

## FUNDING INFORMATION

This study was part funded by grants from the Indian Council of Medical Research (79/159/2015/NCD‐II); National Cancer Grid (2016/001), Tata Consultancy Services Foundation and a Margdarshi Fellowship of the DBT‐Wellcome India‐Alliance (IA/M/12/1/500261). The funding agencies had no role in collection, analyses or interpretation of data.

## CONFLICTS OF INTEREST

The authors declare that they have no competing interests.

## ETHICS APPROVAL STATEMENT

All patients provided written consent for treatment and data analyses at the time of recruitment for the study. Approval for the study was obtained from Tata Medical Center's Institutional Review Board (EC/TMC/12/13).

## Supporting information


Appendix S1

## Data Availability

Anonymized data are available from the corresponding author upon request.
