# Supplementary material for: Comparative treatment costs of risk‐stratified therapy for childhood acute lymphoblastic leukemia in India
Source: Cancer Med. 2022 Aug 15;12(3):3499–508. doi: 10.1002/cam4.5140 (PMC9939102; doi:10.1002/cam4.5140)
Supplement: Supplementary file 1 — Appendix S1 [file CAM4-12-3499-s001.zip › CAM4_5140_Supplementary Data R1_Clean.docx]

**Supplementary Data**

**Table S1**. ALL treatment regimes for the cohort included in this study

| **Risk** | **Induction** | **Consolidation** | **Interim Maintenance** | **Delayed Intensification** | **Maintenace** |
| --- | --- | --- | --- | --- | --- |
| **SR** | P, V, Ax4 | MP | V,De,MP | V,De,DM, A,C, Ara, MP | MP, MTX |
| **IR** | P, V, Ax8, Dx2 | 1B | ivMTX, V | V,De, DM,A,C, Ara, MP | MP, MTX |
| **HR** | P, V, Ax8, Dx4 | Aug 1B | HDMTX, MP | V,De,DM,A,C, Ara, MP | MP, MTX |
| **T** | P, V, Ax8, Dx4 | Aug 1B | HDMTX, MP | V,De,DM,A,C, Ara, MP | MP, MTX |
| SR = Non high risk cytogenetics; prednisolone good response; NCI standard risk; post induction MRD <10-4; CNS1,2 | | | | | |
| IR = Non high risk cytogenetics; prednisolone good response; NCI high risk; post induction MRD <10-4; CNS1,2; bulky disease | | | | | |
| HR = prednisolone poor risk; high risk cytogenetics; MRD ≥10^-4^; CNS3 | | | | |  |
| P = Prednisolone; V = Vincristine; A = Asparaginase; D = Daunorubicin; De = Dexamethasone; MP = 6-Mercaptopurine; | | | | | |
| MTX = Methotrexate; HD = High dose; iv = intravenous; DM = Doxorubicin or Mitoxantrone | | | | | |
| All patients receive intrathecal methotrexate  1B = BFM Consolidation; Aug 1B = Augmented BFM Consolidation  Risk Stratification is reported in Trials. 2022;23(1):102. | | | |  |  |

**Table S2.** Input parameters for DALYs model

| **Model elements** | **Values** | **Source** |
| --- | --- | --- |
| Discount rate | 0.03 (0,0.06) | WHO-CHOICE^1^ |
| India life expectancy, 2019 | 69.7 | World Bank^2^ |
| Mean age at diagnosis | Risk group dependent | Table 1 |
| Duration of disability (length of therapy) | 2.5 | Assumed length of therapy |
| Disability weight during therapy | 0.288 | GBD 2019^3^ |
| Utility score at age 24 y using MEPS^a^ | 0.826 | Yeh et al, JNCI 2016^4^ |
| Utility score at age 35 y using MEPS^a^ | 0.81 | Yeh et al, JNCI 2016^4^ |
| Utility score at age 45 y using MEPS^a^ | 0.791 | Yeh et al, JNCI 2016^4^ |
| Utility score at age 24 y using CCSS survivors^b^ | 0.779 | Yeh et al, JNCI 2016^4^ |
| Utility score at age 35 y using CCSS survivors^b^ | 0.766 | Yeh et al, JNCI 2016^4^ |
| Utility score at age 45 y using CCSS survivors^b^ | 0.753 | Yeh et al, JNCI 2016^4^ |
| No. of new ALL incident cases | 15000 | CanKids, Arora et al, IP 2021^5^ |
| Proportion of patients with 3-year overall survival^c^ | Risk group dependent | Pandey et al, 2021^6^ |
| India GDP per capita, 2019 | 2100.75 | World Bank^7^ |
| Abbreviations: CCSS = Childhood Cancer Survivor Study; GBD = Global Burden of Disease; GDP = Gross Domestic Product | | |
| ^a^MEPS provides utility weights generalizable to the US general population | |  |
| ^b^CCSS provides utility weights for late effects for those who received treatment for cancer in childhood | | |
| ^c^3-year overall survival for HR and T was considered same | |  |

**Table S3.** Cost for chemotherapy for an exemplar patient with body surface area of 1m^2^

|  | **SR** | **IR** | **HR** | **T** |
| --- | --- | --- | --- | --- |
| **Induction** | 127.29 | 235.72 | 247.88 | 245.31 |
| **Consolidation** | 27.16 | 88.18 | 284.39 | 284.39 |
| **Interim Maintenance** | 68.37 | 168.61 | 255.27 | 389.24 |
| **Delayed Intensification** | 164.98 | 164.98 | 164.98 | 164.98 |
| **Maintenance** | 1033.08 | 1033.08 | 1033.08 | 1033.08 |
| **Total** | 1420.88 | 1690.58 | 1985.61 | 2117.00 |
| All costs are in USD ($) | |  |  |  |

**Table S4**. Incremental cost-effectiveness ratio

| **Therapy** | **N** | **Cost** | **Total** | **DALY** | **Cost/DALY** |
| --- | --- | --- | --- | --- | --- |
| **All Purpose** | 15000 | 7400 | 111000000 | 148176.42 | 749.10704 |
| **Risk Stratified** | 15000 | 5880 | 88200000 | 248705.37 | 354.63649 |

All costs are in USD ($)

**Supplemental References for Table S2**

1. Edejer TT, Baltussen R, Adam T, et al. *WHO Guide to Cost-Effectiveness Analysis*. 2003.

2. Bank TW. Life expectancy at birth, total (years) - India. December 2020. <https://data.worldbank.org/indicator/SP.DYN.LE00.IN?end=2019&locations=IN&start=1960>

3. Collaborators GDaI. Global burden of 369 diseases and injuries in 204 countries and territories, 1990-2019: a systematic analysis for the Global Burden of Disease Study 2019. *Lancet*. 10 17 2020;396(10258):1204-1222. doi:10.1016/S0140-6736(20)30925-9

4. Yeh JM, Hanmer J, Ward ZJ, et al. Chronic Conditions and Utility-Based Health-Related Quality of Life in Adult Childhood Cancer Survivors. *J Natl Cancer Inst*. 09 2016;108(9)doi:10.1093/jnci/djw046

5. Arora RS, Bagai P, Bhakta N. Estimated National and State Level Incidence of Childhood and Adolescent Cancer in India. *Indian Pediatr*. May 15 2021;58(5):417-423.

6. Pandey A, Narula G, Kumar S, et al. Primary Outcome of Pediatric B All Patients Treated on ICiCLe Pilot Protocol: Retrospective Analysis from a Tertiary Cancer Center in India. *Pediatric Blood & Cancer*, 2021;68:S124-S124. doi:10.1002/pbc.29349

7. Bank TW. GDP per capita (current US$) - India. December 2021. <https://data.worldbank.org/indicator/NY.GDP.PCAP.CD?locations=IN>
